# Supplementary material for: Alzheimer’s Disease, and Breast and Prostate Cancer Research: Translational Failures and the Importance to Monitor Outputs and Impact of Funded Research
Source: Animals (Basel). 2020 Jul 14;10(7):1194. doi: 10.3390/ani10071194 (PMC7401638; doi:10.3390/ani10071194)
Supplement: Supplementary file 1 [file animals-10-01194-s001.pdf]

Table S1. Currently available mouse models of Alzheimer's disease (retrieved from: https://www.alzforum.org/research-models/alzheimers-disease).

Name	Modifications	Neuropathology
3xTg	APP: Transgenic; PSEN1: Transgenic; MAPT: Transgenic	Age-related, progressive neuropathology including plaques and tangles. Extracellular Aβ deposits by 6 months in frontal cortex, more extensive by 12 months. No tau pathology at 6 months, but evident at 12 months. Synaptic dysfunction, including LTP deficits, prior to plaques and tangles.
5xFAD (B6SJL)	APP: Transgenic; PSEN1: Transgenic	Amyloid pathology starting at 2 months, including amyloid plaques. Accumulation of intraneuronal Aβ before amyloid deposition. Gliosis and synapse degeneration. Neuron loss in cortical layer 5 and subiculum. No neurofibrillary tangles.
5xFAD (C57BL6)	APP: Transgenic; PSEN1: Transgenic	Amyloid pathology starting at 2 months, including amyloid plaques. Accumulation of intraneuronal Aβ before amyloid deposition. Gliosis and synapse degeneration. Neuron loss in cortical layer V.
A7 APP transgenic	APP: Transgenic	Progressive amyloid deposition in the cerebral cortex by approximately 9-12 months.
Abca7*A1527G/APOE4/Trem2*R47H	Abca7: Knock-In; APOE: Knock-In; Trem2: Knock-In	Unknown.
Abca7 KO/APOE4/Trem2*R47H	Abca7: Knock-Out; APOE: Knock-In; Trem2: Knock-In	Unknown.
ADanPP	ITM2B (BRI2): Transgenic	ADan deposition starts in the hippocampus and meningeal vessels at 2 months and increases with age. By 18 months, deposition is widespread. The majority of amyloid deposits are associated with the vasculature, where they destroy the integrity of the vessel wall and lead to microhemorrhages. Parenchymal amyloid plaques surrounded by microglia and dystrophic neurites are also present.
AD-BXD	APP: Transgenic; PSEN1: Transgenic	Transgenic AD-BXD mice develop amyloid plaques by 6 months of age, although the extent of plaque deposition is strain-dependent.
APLP2 Knock-out	Aplp2: Knock-Out	Not observed.
APOE2 Knock-In	APOE: Knock-In	Unknown.
APOE2 Knock-In, floxed (CureAlz)	APOE: Knock-In	Unknown.
APOE2 Knock-In (JAX)	APOE: Knock-In	Unknown.
APOE2 Targeted Replacement	APOE: Knock-In	Unknown.
APOE3 Knock-In, floxed (CureAlz)	APOE: Knock-In	Unknown.
APOE3 Knock-In (JAX)	APOE: Knock-In	No data.
APOE3 Knock-In (Lamb)	APOE: Knock-In	Unknown.
APOE3 Targeted Replacement	APOE: Knock-In	Unknown.
APOE4 Knock-In, floxed (CureAlz)	APOE: Knock-In	Unknown.
APOE4 Knock-In (JAX)	APOE: Knock-In	No data.
APOE4 Knock-In (Lamb)	APOE: Knock-In	Unknown.
APOE4 Targeted Replacement	APOE: Knock-In	Unknown.
APOE Knock-out	APOE: Knock-Out	Unknown.

APP23	APP: Transgenic	Aβ deposits first observed at 6 months. Congophilic plaques increase in size and number with age and are surrounded by activated microglia, astrocytes, and dystrophic neurites containing hyperphosphorylated tau (although no neurofibrillary tangles). Neuronal loss in the CA1 region of the hippocampus. Mice also develop CAA, and microhemorrages occur at later ages.
APP23 x PS1-R278I	APP: Transgenic; PSEN1: Knock-In	Amyloid deposition by 6 months of age in the cortex and hippocampus. Abundant reactive astrocytes in the vicinity of plaques. Elevated Aβ43 in the brain by 3 months. High density of cored plaques. Pyroglutamate Aβ (N3pE-Aβ) associated with amyloid plaques.
APP751SL/PS1 KI	APP: Transgenic; PSEN1: Knock-In	Acceleration of extracellular Aβ deposition compared to the single transgenics. Age-dependent neuronal loss in the hippocampus with extensive neuronal loss in the CA1/2 at 10 months with detection as early as 6 months in female mice. Intraneuronal Aβ and thioflavin-S-positive deposits before neuronal loss. Astrogliosis in proximity of Aβ-positive neurons.
APP-C99 (tg13592)	APP: Transgenic	No neuropathology up to age 29 months; however, pathology reminiscent of inclusion body myopathy observed at 6-12 months: Aβ-immunoreactive deposits in skeletal muscle fibers. Muscle fibers with Aβ-immunoreactive deposits increased with age and also became vacuolated.
APPDutch	APP: Transgenic	Increased Aβ40/42 ratio. Extensive vascular Aβ deposition starting at 22-24 months appearing first in leptomeningeal vessels followed by cortical vessels, leading to smooth muscle cell degeneration, hemorrhages, and neuroinflammation. Parenchymal amyloid plaques are not observed.
APP E693Δ-Tg (Osaka)	APP: Transgenic	Age-dependent accumulation of Aβ oligomers within hippocampal and cortical neurons, but negligible deposits of extracellular amyloid. Abnormal tau phosphorylation, but no overt tangle pathology. Synaptic loss and gliosis in hippocampus and cerebral cortex. Late neuronal loss in the CA3 region of the hippocampus.
APP Knock-in	APP: Knock-In	Unknown.
APP Knock-out	APP: Knock-Out	Elevated reactive gliosis by 14 weeks in the hippocampus and parts of the neocortex.
App KO/APOE4/Trem2*R47H	APOE: Knock-In; App: Knock-Out; Trem2: Knock-In	Unknown.
APP NL-F Knock-in	APP: Knock-In	Elevated Aβ peptides accumulating into plaques starting at 6 months. Microgliosis and astrocytosis, especially around plaques. Reduced synaptophysin and PSD-95 indicative of synaptic loss. No tangle pathology or neurodegeneration.
APP NL-G-F Knock-in	APP: Knock-In	Aggressive amyloidosis with deposition in the cortex beginning at 2 months and approaching saturation by 7 months. Aβ deposition in heterozygous mice at 4 months. Subcortical amyloidosis. Exacerbated microgliosis and astrocytosis compared to APP^{NL-F} mice. Reduced synaptophysin and PSD-95 indicative of synaptic loss. No tangle pathology or neurodegeneration.
AppNL-G-F/MAPT double knock-in	App: Knock-In; MAPT: Knock-In	Amyloid plaques, plaque-associated neuritic dystrophy, and neuroinflammation, similar to App^{NL-G-F}.
APPPS1	APP: Transgenic; PSEN1: Transgenic	Amyloid plaque deposition starts at approximately 6 weeks in the neocortex. Amyloid deposits in the hippocampus appear at 3-4 months, and in the striatum, thalamus and brainstem at 4-5 months. Phosphorylated tau-positive neuritic processes have been observed in the vicinity of all congophilic amyloid deposits, but no fibrillar tau inclusions are seen.

APP/PS1/rTg21221	APP: Transgenic; PSEN1: Transgenic; MAPT: Transgenic	Tau accumulations, dystrophic neurites, astrocytosis, neuronal loss, and synapse loss were more pronounced adjacent to cortical plaques. Tangles were not observed.
APPsw/0; Pdgfrβ+/-	APP: Transgenic; PDGFRB: Knock-Out	Amyloid plaques; elevated brain interstitial human and murine Aβ due to reduced clearance of soluble Aβ, cerebral amyloid angiopathy, tau hyperphosphorylation and related pathology. Neurite loss and neuronal loss in the cortex and hippocampus.
APPSwDI x NOS2 Knock-out	APP: Transgenic; NOS2: Knock-Out	Plaques especially in the thalamus and subiculum. Aggregated, hyperphosphorylated tau tangles. Neuronal loss especially of NPY neurons in the hippocampus and subiculum. More severe pathology than Tg-SwDI alone.
APPSwe	APP: Transgenic	Amyloid plaques by 17-18 months in the neocortex and hippocampus with detection of 5-10 fold more Aβ40 than Aβ42. Plaque burden significantly lower than in the double transgenic PS2APP. Lower levels of insoluble Aβ40 and Aβ42 than the PS2APP mouse at 16-18 months.
APP(Swedish) (R1.40)	APP: Transgenic	By 14-16 months, homozygotes have diffuse and compact Aβ deposits in the frontal cortex, by 18-20 months plaques throughout the cortex and olfactory bulb with occasional deposits in the corpus callosum and hippocampus. No tangles, but some changes in phosphorylated tau. Reactive astrocytes and microglia by 14-16 months.
APPSwe (line C3-3)	APP: Transgenic	Age-associated increase in Aβ40 and Aβ42 and some amyloid deposition at advanced age.
APPSwe (line E1-2)	APP: Transgenic	Age-dependent increase in Aβ42, with low levels at 6-14 months and high levels at 24-26 months.
APPSweLon	APP: Transgenic	No amyloid plaques observed at 2 years.
APPSwe/PSEN1(A246E)	APP: Transgenic; PSEN1: Transgenic	Amyloid plaques by 9 months, starting in the hippocampus and subiculum. Plaques later develop in the cortex; the striatum and thalamus are relatively spared. Amyloid pathology is more severe in females. Dystrophic neurites and gliosis in the cortex and hippocampus.
APPSwe/PSEN1dE9 (C3-3 x S-9)	APP: Transgenic; PSEN1: Transgenic	Elevated Aβ42 and plaques in the hippocampus and cortex. No tangles. Reduced cholinergic markers.
APPswe/PSEN1dE9 (C57BL6)	APP: Transgenic; PSEN1: Transgenic	Amyloid plaques begin to emerge in the cortex at about 4 months of age and in the hippocampus at about 6 months. Gliosis and dystrophic neurites are associated with plaques. Amyloid angiopathy has been observed in the retina.
APPswe/PSEN1dE9 (line 85)	APP: Transgenic; PSEN1: Transgenic	Occasional Aβ deposits by 6 months with abundant plaques in the hippocampus and cortex by 9 months and a progressive increase in plaques up to 12 months. No tangles. Decrease in synaptic markers and increase in complement immunoreactivity.
APPSw-NSE	APP: Transgenic	Increased Aβ42 in the cortex and hippocampus of 12 month old mice, but no plaques. Increased tau phosphorylation and TUNEL-stained nuclei relative to control mice.
APP(V642I)KI	APP: Transgenic	Increased Aβ42(43) relative to Aβ40 at 29 months, but without neuritic plaques, neurofibrillary tangles, massive neuronal loss, or brain atrophy.
APP(V717I)	APP: Transgenic	Plaques start in the subiculum, spreading to the frontal cortex as dense and diffuse aggregates. Prominent amyloid deposits in brain vessels after 15 months. Microbleeds. Amyloid-associated inflammation. CSF Aβ42/Aβ40 ratio decreases from 15 months. Dystrophic neurites containing hyperphosphorylated tau, but no tangle pathology.

APP(V717I) x PS1(A246E)	APP: Multi-transgene; PSEN1: Transgenic	Soluble, oligomeric Aβ at 2 months and increases with age. Amyloid plaques at 6-9 months, earlier than APP(V717I) single transgenics. Plaques start in the subiculum and spread to the frontal cortex. Amyloid-associated inflammation. CAA pathology at 8 months; microbleeds at 12-15 months. Dystropic neurites containing hyperphosphorylated tau, but no tangle pathology.
Arc48 (APPSw/Ind/Arc)	APP: Transgenic	Parenchymal neuritic plaques by 2 months accompanied by dystrophic neurites. Prominent hippocampal Aβ deposition by 3-4 months. Relatively low Aβ42/Aβ40 ratio. Comparable cerebrovascular amyloid deposition to J20.
ArcAβ	APP: Transgenic	At 6 months intracellular punctate deposits of Aβ abundant in cortex and hippocampus, but overt β-amyloid plaques not apparent until 9-15 months. Severe CAA also present at this age with dense Aβ aggregates in blood vessels walls and spreading into the parenchyma.
ARTE10	APP: Transgenic; PSEN1: Transgenic	Robust early plaque development (by 3 months in homozygotes, 5 months in hemizygotes), predominantly congophilic dense-core amyloid plaques surrounded by dystrophic neurites and gliosis. Some diffuse plaques and cerebral amyloidosis. No tau tangles. Neurons have reduced dendritic length, surface area, and branches.
BACE1 cKO (Hu, Yan) X 5xFAD	Bace1: Conditional Knock-out; APP: Transgenic; PSEN1: Transgenic	Amyloid plaques, reactive astrocytes and microglia, and dystrophic neurites accumulate up to day 120, but to a lesser degree than in control 5xFAD (5xFAD mice homozygous for a floxed Bace1 gene), then recede thereafter.
Bace1 conditional knock-out (adult, whole body) (Vassar)	Bace1: Conditional Knock-out	Defects in axonal organization.
BACE1 conditional knock-out (Hu, Yan)	Bace1: Conditional Knock-out	No gross morphological changes observed.
Bace1 conditional knock-out (neonatal, forebrain) (Vassar)	Bace1: Conditional Knock-out	Hypomyelination and defects in axon organization.
Bace1 conditional knockout (Tesco)	Bace1: Conditional Knock-out	None observed: hippocampal mossy fiber organization and sciatic-nerve myelination were normal.
BACE1 Knock-out	BACE1: Knock-Out	Hypomyelination in the hippocampus and cerebral cortex, but normal axonal development.
BACE2 Knock-out	BACE2: Knock-Out	Not observed.
BRI-Aβ40 (BRI2-Aβ40)	Transgenic	No overt amyloid pathology or detergent-insoluble amyloid-β.
BRI-Aβ42 (BRI2-Aβ42)	Transgenic	Detergent-insoluble amyloid-β appearing with age and cored plaques as early as 3 months in the cerebellum. Variable forebrain pathology later with extracellular Aβ plaques in the hippocampus and entorhinal/piriform cortices at 12 months. Age-associated congophillic amyloid angiopathy. No tangles or neuronal loss.
CAST.APP/PS1	APP: Transgenic; PSEN1: Transgenic	Amyloid plaques, plaque-associated gliosis, cerebral amyloid angiopathy; possible neuron loss in hippocampal area CA1.
Ceacam1 KO/APOE4/Trem2*R47H	APOE: Knock-In; Ceacam1: Knock-Out; Trem2: Knock-In	Unknown.

Clasp2*L163P/APOE4/Trem2*R47H	Clasp2: Knock-In; APOE: Knock-In; Trem2: Knock-In	Unknown.
CNS-restricted PS1 cKO	PSEN1: Conditional Knock-out	Premature differentiation of neural progenitor cells results in reduced cells and neurons. 45 percent of late-born neurons fail to migrate to their appropriate positions in the superficial cortical layers.
E2FAD	APOE: Knock-In; APP: Transgenic; PSEN1: Transgenic	Amyloid plaques starting at 4 months and increasing with age. Gliosis and loss of synaptic proteins.
E3FAD	APOE: Knock-In; APP: Transgenic; PSEN1: Transgenic	Amyloid plaques starting at 4 months and increasing with age. Gliosis and loss of synaptic proteins.
E4FAD	APOE: Knock-In; APP: Transgenic; PSEN1: Transgenic	Amyloid plaques starting at 4 months and increasing with age. Gliosis and loss of synaptic proteins.
GFAP-APOE4/APOE Knock-out	APOE: Transgenic; APOE: Knock-Out	Developing and adult mice express human APOE4 in glia and neuropil.
hAbeta-loxP-KI	APP: Knock-In	Unknown.
hAPP/APOE4/Trem2*R47H	APOE: Knock-In; APP: Knock-In; Trem2: Knock-In	Unknown.
hBACE	BACE1: Transgenic	No evidence of Aβ deposition in single transgenic animals.
hBACE54	BACE1: Transgenic	Not observed.
hCR1 KI on APOE4/Trem2	Cr2: Knock-Out; CR1: Knock-In; CR2: Knock-In; APOE: Knock-In; Trem2: Knock-In	Unknown.
htau	MAPT: Knock-Out; MAPT: Transgenic	Age-associated tau pathology, including redistribution of tau to cell bodies and dendrites, phosphorylated tau, accumulation of aggregated paired helical filaments, and ultimately thioflavin-S positive neurofibrillary tangles. Pathology most severe in neocortex and hippocampus, and minimal in the brain stem and spinal cord. Some neuronal loss.
hTau-A152T	MAPT: Transgenic	Tangles or dense tau inclusions not observed. Abnormal accumulations of soluble tau. Age-dependent neuronal loss was observed in the hippocampus.
hTau-AT (hTau40-AT)	MAPT: Knock-In	Tangles in hippocampus, cortex, and spinal cord at 3 months with age-dependent increases. Tau hyperphosphorylation, conformation changes, and mislocalization observed. Age-dependent loss of synapses.
hTau.P301S	MAPT: Transgenic	Age-dependent hyperphosphorylation of tau and conformational changes leading to neurofibrillary tanglelike pathology in the cerebral cortex, hippocampus, brain stem, and spinal cord. Neurodegeneration, especially in the spinal cord, accompanied by astrocytosis.
Human-BACE1	BACE1: Transgenic	Not observed.

Il1rap KO/APOE4/Trem2*R47H	APOE: Knock-In; Il1rap: Knock-Out; Trem2: Knock-In	Unknown.
J20 (PDGF-APPSw,Ind)	APP: Transgenic	Age-dependent formation of Aβ plaques. Dystrophic neurites associated with plaques. No tangles. Variable cell loss. Decrease in synaptic markers and increase in complement immunoreactvity.
JNPL3(P301L)	MAPT: Transgenic	Age and gene-dose dependent development of neurofibrillary tangles as early as 4.5 months in homozygotes and 6.5 months in heterozyotes. Tangles and Pick-body-like inclusions in the amygdala, hypothalamus, pons, medulla, and spinal cord among other areas. Neuronal loss, especially in the spinal cord.
Kif21b*T82T/APOE4/Trem2*R47H	Kif21b: Knock-In; APOE: Knock-In; Trem2: Knock-In	Unknown.
MAPT knock-in	MAPT: Knock-In	No evidence of increased neuroinflammation, neuronal death, or brain atrophy in MAPT knock-in mice, compared with wild-type mice.
Mthfr*C677T/APOE4/Trem2*R47H	Mthfr: Knock-In; APOE: Knock-In; Trem2: Knock-In	Unknown.
mThy-1 3R Tau (line 13)	MAPT: Transgenic	Accumulation of 3R tau in neurons of the cortex and hippocampus. Pick body-like tau aggregates and neuronal loss in the hippocampus and cortex. Astrogliosis, with some 3R tau in GFAP-positive astrocytes. Synapto-dendritic changes and mitochondrial pathology.
mThy1-hAPP751 (TASD41)	APP: Transgenic	Age-dependent increases in Aβ40 and Aβ42, with Aβ42 > Aβ40. Plaques at an early age, starting at 3-6 months in the frontal cortex. At 5-7 months, size and number of plaques increased in the frontal cortex, and dense amyloid deposits appear in hippocampous, thalamus, and olfactory region.
NSE-ApoE3	APOE: Transgenic	Human ApoE3 protected against the age-dependent neurodegeneration seen in APOE -/- mice.
NSE-ApoE4	APOE: Transgenic	Not observed.
NSE-APP751	APP: Transgenic	Age-dependent increase in Aβ deposits and tau immunoreactivity.
NSE-hPS2(N141I)	PSEN2: Transgenic	Not observed.
PDAPP(line109)	APP: Transgenic	Amyloid plaques in the hippocampus, cerebral cortex. Gliosis. Dystrophic neurites. Decreased synaptic and dendritic density in the hippocampus.
PDGF-APPSw,Ind (line J9)	APP: Transgenic	Amyloid plaques at 8-10 months, but not at 2-4 months when deficits in synaptic transmission are observed. Approximately 20% of mice had plaques at 5-7 months, 50% at 8-10 months, and 100% by 21-25 months.
PDGF-APP(WT) (line I5)	APP: Transgenic	Expression of human APP in the brain especially in the neocortex and hippocampus. No plaques up to 24 months.
PLB1-triple (hAPP/hTau/hPS1)	APP: Multi-transgene; MAPT: Multi-transgene; PSEN1: Multi-transgene	Age-related neuropathology including intraneuronal and oligomeric Aβ accumulation and hyperphosphorylated tau in the hippocampus and cortex from six months. Minimal amyloid plaques up to 21 months. Subtle tau pathology, but no overt tangles. Cortical hypometabolism with increased metabolic activity in basal forebrain and ventral midbrain by FDG-PET/CT.

PLB4 (hBACE1)	BACE1: Transgenic	Elevated extracellular multimeric Aβ, including Aβ*56 and Aβ hexamers, in the absence of plaques. At 12 months of age, astrogliosis was observed in a region- and genotype-dependent manner, especially in the dentate gyrus, hippocampal CA1, and piriform cortex. No overt tau pathology.
Plcg2 KO	Plcg2: Knock-Out	Unknown.
Plcg2*M28L/APOE4/Trem2*R47H	APOE: Knock-In; Plcg2: Knock-In; Trem2: Knock-In	Unknown.
Prnp-APP	APP: Transgenic	Unknown.
PS1(A246E)	PSEN1: Transgenic	Histologically normal up to 2 years old by hematoxylin-eosin, silver, and thioflavin-S staining.
PS1 conditional Knock-out	PSEN1: Conditional Knock-out	Reduction in Aβ40 and Aβ42 peptides; accumulation of APP C-terminal fragments.
PS1(M146L)	PSEN1: Transgenic	No abnormal pathology up to 2.5 years. Elevated Aβ2(43); no effect on Aβ40. Altered mitochondrial activity. Disregulation of calcium homeostasis.
PS1(M146V)	PSEN1: Transgenic	No abnormal neuropathology up to 2.5 years. Elevated Aβ42(43). Altered mitochondrial activity and disregulation of calcium homeostasis.
PS1 P264L	PSEN1: Knock-In	Not observed.
PS2APP	APP: Transgenic; PSEN2: Transgenic	Age-associated development of plaques: none at 3 months, overt Aβ deposition in the brain at approximately 6 months, with heavy plaque load in the hippocampus, frontal cortex, and subiculum at 10 months. Aβ deposits in blood vessels were sporadic, mainly in large vessels. Cerebral amyloid deposits correlate with levels of the human APP transcript at 12 months.
PS2APP (PS2(N141I) x APPswe)	APP: Transgenic; PSEN2: Transgenic	Rare amyloid deposits at 5 months, with consistent deposits in the subiculum and frontolateral cortices by 9 months. Plaques increase in number and distribution with time, spreading throughout the neocortex and hippocampus as well as the amygdala and thalamic and pontine nuclei. The distribution and abundance of activated microglia and astrocytes correlate with Aβ deposition.
PS2(N141I)	PSEN2: Transgenic	Unknown.
PS/APP	APP: Transgenic; PSEN1: Transgenic	Aβ accumulates in the cerebral cortex and hippocampus starting ~6 months and increasing with age. Other regions affected later. Deposition occurs in white matter, cerebrovasculature, and grey matter in the form of diffuse and fibrillar plaques. Fibrillar deposits are associated with dystrophic neurites and GFAP-positive astrocytes at ~ 6 months with later microglial activation.
PS cDKO	PSEN1: Conditional Knock-out; PSEN2: Knock-Out	At 2 months the number of apoptotic neurons is elevated about 8-fold. By 6 months, about 18 percent of of cortical neurons are lost. Up-regulation of inflammatory markers and progressive astrogliosis and microgliosis in the neocortex and hippocampus.
PSEN1-flox	PSEN1: Knock-Out	No morphological abnormalities. When crossed with Cre recombinase driven by Thy1, brain levels of Aβ40 and Aβ42 decrease and C-terminal fragments of APP accumulate.
PSEN1 Knock-out	PSEN1: Knock-Out	Impaired neurogenesis. Massive neuronal loss. Hemorrhages in the CNS.
PSEN1(M146V) Knock-In	PSEN1: Knock-In	Hypersensitive to kainate-induced degeneration and death of CA3, CA1 and hilar neurons. Cultured hippocampal neurons have increased vulnerability to death induced by glutamate. Disrupted calcium homeostasis. Increased oxidative stress and mitochondrial dysfunction.
PSEN1(P117L) (line 13)	PSEN1: Transgenic	No plaques or diffuse amyloid deposits at 2-3 months. Elevated generation of Aβ42.

PSEN1(WT)	PSEN1: Transgenic	No pathological changes have been observed in these mice.
PSEN1-YAC (line G9)	PSEN1: Transgenic	Elevated Aβ42 in the brain and plasma. Higher levels and earlier Aβ deposition when crossed with APP YAC line R1.40.
PSEN2 Knock-out	PSEN2: Knock-Out	No gross brain abnormalities or astrogliosis.
PWK.APP/PS1	APP: Transgenic; PSEN1: Transgenic	Amyloid plaques and plaque-associated gliosis by 8 months.
rTg9191	APP: Transgenic	Age-associated pathology in the cerebral cortex and hippocampus starting at 8 and 10½-12½ months of age, respectively. Gliosis and hyperphosphorylated tau in the vicinity of dense-core plaques. Fibrillar oligomeric species, e.g., Aβ dimers.
rTgTauEC	MAPT: Transgenic	Propagating tau pathology starting in the entorhinal cortex and spreading to regions functionally connected to the EC (e.g., dentate gyrus). Neurodegeneration and axonal degeneration, first in EC and parasubiculum. Gliosis and synaptic loss.
rTg(tauP301L)4510	MAPT: Transgenic	Argyrophilic tangle-like inclusions in cortex by 4 months and in hippocampus by 5.5 months. Decreased CA1 neurons (~60 percent) by 5.5 months. Gross forebrain atrophy by 10 months. The number of CA1 neurons stabilized after a brief (six to eight week) suppression of transgenic tau.
Senescence Accelerated Mouse (SAMP8)	Spontaneous	Age-associated increase in hippocampal Aβ from 4 to 12 months, but no plaque-like structures by Congo red or thioflavine S. Spongiform degeneration: vacuoles of various size in the neuropil in the brain stem. Microglial cell proliferation. Degeneration of dopamine neurons in the substantia nigra and noradrenaline neurons in the locus coeruleus.
Snx1*D465N/APOE4/Trem2*R47H	Snx1: Knock-In; APOE: Knock-In; Trem2: Knock-In	Unknown.
Sorl1*A528T/APOE4/Trem2*R47H	Sorl1: Knock-In; APOE: Knock-In; Trem2: Knock-In	Unknown.
TAS10 (thy1-APPswe)	APP: Transgenic	Age-related accumulation of Aβ in the hippocampus and cortex leading to plaque deposition by 12 months of age. Early gliosis and dystrophic neurites, not limited to the vicinity around plaques. Changes in synaptic morphology and number, along with increased number of lysosomes.
TASTPM (TAS10 x TPM)	APP: Transgenic; PSEN1: Transgenic	Aβ deposits beginning at 3 months of age, with fibrillar plaques by 6 months in the cerebral cortex and hippocampus. Some vascular amyloid. Plaques surrounded by dystrophic neurites and reactive glia. No tangles or neuronal loss. Female mice have more rapid and severe amyloid pathology.
Tau264	MAPT: Transgenic	No overt neuropathology even at the advanced age of 24 months.
Tau35	MAPT: Transgenic	Progressive tau pathology in the hippocampus, including abnormally phosphorylated and misfolded tau, mislocalized tau, and tangle-like structures. Dystrophic neurites.
Tau4RTg2652	MAPT: Transgenic	Extensive pretangle pathology throughout the brain (e.g. phospho- tau) but no mature neurofibrillary tangles and only mild oligomeric tau, restricted to the CA1 region of the hippocampus. Dystrophic neurites and axonal pathology (spheroids). No overt neuronal loss.
Tau609 (Tau 10 + 16)	MAPT: Transgenic	Aggregated tau in neurons of the entorhinal cortex, hippocampus, and cerebral cortex at advanced ages. Intraneuronal accumulation of tau oligomers in the hippocampus. Neuronal loss in the

		entorhinal cortex and hippocampus. Gliosis. Some hippocampal areas affected by age-related synaptic dysfunction and reduced synaptic density.
TauA152T-AAV	MAPT: Virus	Neuron loss and astrogliosis were observed in the cortices of 3-month-old mice.
TauC3 (Transgenic caspase-cleaved tau)	MAPT: Transgenic	No significant cell loss or astrogliosis in the brain. Age-dependent reduction in synaptic proteins (e.g. synaptophysin, PSD95) by 1.3 to 3 months of age. Hyperphosphorylated tau oligomers and aggregates.
Tau Exon 10 Knock-out	MAPT: Knock-Out	No overt neuropathology at 12 months of age.
TauΔK280 ("Proaggregation mutant")	MAPT: Transgenic	Abundant pre-tangle pathology, but only rare mature tangles, and only at advanced ages. Tau pathology included mislocalization of tau to the somatodendritic compartment, aggregation, and hyperphosphorylation.
Tau P301L	MAPT: Transgenic	Pathologic hyperphosphorylation and conformational change of parenchymal tau in brain tissues starting at 7 months. Tangle-like pathology is mainly observed in the brain stem and spinal cord, and to a lesser extent in the midbrain and cerebral cortex. Age-dependent increase in total tau in CSF.
TauP301L-AAV	MAPT: Virus	Neurofibrillary tangles and gliosis, but no cortical neuron loss, at 6 months of age.
Tau P301S (Line PS19)	MAPT: Transgenic	Neuron loss and brain atrophy by eight to 12 months, especially in the hippocampus and spreading to the neocortex and entorhinal cortex. Neurofibrillary tangles in the neocortex, amygdala, hippocampus, brain stem, and spinal cord. Neuroinflammation with microgliosis and astrocytosis.
TauPS2APP	APP: Transgenic; MAPT: Transgenic; PSEN2: Transgenic	Phosphorylated tau accumulation in the subiculum and the CA1 region of the hippocampus at 4 months. Neurofibrillary tangles in these regions as well as the amygdala. Amyloid plaques. Dystrophic neurites and neuropil threads containing abnormally phosphorylated tau. No overt neuronal loss.
Tau R406W transgenic	MAPT: Transgenic	Argyrophilic and congophilic tau inclusions in neurons of the forebrain with age. Detectable with Congo red, thioflavin-S and Gallyas silver stain. Congophilic tau inclusions also in the hippocampus and amygdala. Mainly straight tau filaments.
TauRDΔK280 (“Proaggregation mutant”)	MAPT: Transgenic	Tau aggregates and tangles as early as 2-3 months after gene expression. Gallyas silver-positive neurons abundant in the entorhinal cortex and amygdala, spreading to the neocortex by 15 months. “Ballooned” neurons. Astrogliosis. Synaptic structural changes and reduced synaptic number. Hippocampal neuronal loss.
Tau V337M	MAPT: Transgenic	SDS-insoluble tau aggregates in hippocampus. Degenerating neurons in the hippocampus containing phosphorylated and ubiquitinated tau aggregates with β-sheet structure.
TBA42	APP: Transgenic	Intraneuronal accumulation of Aβ peptides in the hippocampus by 3 months and in cerebellar nuclei by 6 months. Marked gliosis in the hippocampus by 12 months. Very rare extracellular Aβ deposits.
TetO-APPSweInd (line 102)	APP: Transgenic	APP protein 10-30x higher than endogenous mouse APP. Progressive amyloid plaques starting at 2 months. Extensive amyloid pathology by 9 months especially in the cortex and hippocampus. Amyloid pathology is halted by transgene suppression but existing plaques are stable. Highest doxycycline sensitivity relative to lines 107 and 885.
TetO-APPSweInd (line 107)	APP: Transgenic	APP protein 10-30x higher than endogenous mouse APP. Progressive amyloid plaques starting at 2 months. Extensive amyloid pathology by 9 months especially in the cortex and hippocampus.

		Amyloid pathology is halted by transgene suppression but existing plaques are stable. Intermediate expression of transgene and doxycycline sensitivity relative to lines 102 and 885.
TetO-APPSweInd (line 885)	APP: Transgenic	APP protein 10-30x higher than endogenous mouse APP. Progressive amyloid plaques starting at 2 months. Extensive amyloid pathology by 9 months especially in the cortex and hippocampus. Amyloid pathology is halted by transgene suppression but existing plaques are stable. Highest transgene expression and highest doxycycline requirement relative to lines 102 and 107.
Tg2576	APP: Transgenic	Numerous parenchymal Aβ plaques by 11-13 months with some vascular amyloid. Oxidative lipid damage, astrogliosis and microgliosis. No tangles or neuronal loss.
Tg2576/Tau(P301L) (APPSwe-Tau)	APP; MAPT: Transgenic	Gradual appearance of plaques; by 9 months plaques are scattered throughout the cortex, hippocampus, and amygdala similar to Tg2576. Tau pathology more extensive than JNPL3. Astrocytosis and microgliosis.
Tg4-42	APP: Transgenic	Aβ4-42 is dectable starting at two months, predominantly in the CA1 region of the hippocampus, but also in the occipital cortex, piriform cortex, striatum, and superior colliculus. Age- and dose-dependent hippocampal neuronal loss is seen in the CA1 region as well as microgliosis and astrogliosis.
TgAPParc	APP: Transgenic	Mild amyloid pathology with a relatively late onset, starting with intracellular Aβ, then diffuse extracellular Aβ deposits in the subiculum, expanding to interconnected brain regions such as retrosplenial granular cortex, thalamus, and mammillary bodies. Pathology more severe in females.
tg-APPSwe	APP: Transgenic	Extracellular amyloid deposition begins at ~12 months. Intraneuronal Aβ aggregates at ~6 months. Extracellular pathology, both cerebrovascular amyloid angiopathy (CAA) and congophilic parenchymal plaques, mainly found in the cerebral cortex, hippocampus and thalamus. Aβ-burden in cerebral cortex is approximately 1.0% (at 12 months) and 2.8% (at 18 months).
TgAPPSwe-KI	APP: Knock-In	Accumulation of human Aβ.
Tg-ArcSwe	APP: Transgenic	Strong intraneuronal Aβ aggregation starting at 1 month and increasing with age. Extracellular amyloid plaque at 5-6 months, most consistent in the cerebral cortex, hippocampus, and thalamus. Congophilic parenchymal plaques are predominant, but some mice show marked CAA, particularly in the thalamus.
TgCRND8	APP: Transgenic	Rapid, early plaque development, with thioflavin S-positive amyloid deposits at 3 months; dense cored plaques and neuritic pathology by 5 months. Plaques become more extensive with age. More Aβ42 than Aβ40. Activated microglia appear concurrently with plaques, whereas GFAP+ astrocytes follow later, about 13-14 weeks. Dystrophic neurites at 5 months .
Tg-FDD	ITM2B (BRI2): Transgenic	Widespread cerebral amyloid angiopathy (CAA) starting around 7 months. Deposition of the Danish amyloid subunit (ADan) in brain parenchyma and vessels, along with amyloid-associated gliosis and inflammation, intracellular and extracellular deposition of oligomeric ADan, and tau-positive deposits in neuropil, but no neurofibrillary tangles.
Tg-mAPP/DN-RAGE	APP: Transgenic; RAGE (AGER): Transgenic	Diminished neuropathology compared with mice expressing mutant APP alone at both 3–4 and 14–18 months of age.
Tg-mAPP/RAGE	APP: Transgenic; RAGE (AGER): Transgenic	Increased activation of microglia and astrocytes compared to mice expressing mutant APP alone.

Tg-SwDI (APP-Swedish,Dutch,Iowa)	APP: Transgenic	Hemizygotes progressively accumulate insoluble Aβ40 and Aβ42, especially within brain microvessels starting at 3 months. Fibrillar Aβ in micovessels around 6 months. Diffuse plaque-like deposits around 3 months in the subiculum, hippocampus and cortex. Aβ deposits throughout the forebrain by 12 months.
THY-Tau22	MAPT: Transgenic	A variety of tau pathologies starting at 3 months, including neurofibrillary tangle-like inclusions, rare ghost tangles, and paired helical filament-like structures. Hyperphosphorylation of tau on many epitopes (e.g. AT8, AT100, AT180, AT270, 12E8, tau-pSer396, and AP422) and mild astrogliosis.
TMHT (Thy-1 mutated human tau)	MAPT: Transgenic	Increased total tau, and phosphorylated tau (Thr181, Ser199, Thr231) in amygdala and hippocampus starting at 3 months.
TPM (Thy-1 PS1.M146V)	PSEN1: Transgenic	No plaques.
TREM2-BAC	TREM2: Transgenic	No obvious neuropathology is observed at 4, 7 and 11 months of age.
TREM2-BAC X 5xFAD	TREM2: Transgenic; APP: Transgenic; PSEN1: Transgenic	Amyloid plaques with plaque-associated microgliosis. Reduced plaque burden, altered microglial and plaque morphology, and less severe plaque-associated neuritic dystrophy, compared with 5xFAD.
Trem2 flox	Trem2: Knock-In	No data.
TREM2, humanized (common variant) X 5XFAD	Trem2: Knock-Out; TREM2: Transgenic; APP: Transgenic; PSEN1: Transgenic	Amyloid plaques surrounded by activated microglia.
TREM2, humanized (R47H) X 5XFAD	Trem2: Knock-Out; TREM2: Transgenic; APP: Transgenic; PSEN1: Transgenic	Lower density of activated microglia surrounding amyloid plaques in 5XFAD mice expressing the R47H variant of human TREM2 compared with those expressing the common variant.
Trem2 KO (Colonna)	Trem2: Knock-Out	Microglial number remains constant and microglial size decreases with age in the corpus callosum of Trem2 KO mice, while microglial number increases and microglial size remains stable in wild-type mice.
Trem2 KO (Colonna) x 5XFAD	Trem2: Knock-Out; APP: Transgenic; PSEN1: Transgenic	Compared with 5XFAD, mice deficient in TREM2 show an age- dependent increase in amyloid accumulation in the hippocampus, more severe plaque-associated neuritic dystrophy, and exaggerated neuron loss in the cortex. Microglial containment of plaques is compromised in TREM2-deficient animals. Microglia accumulate autophagosomes.
Trem2 KO (Colonna) x PS19	Trem2: Knock-Out; MAPT: Transgenic	Microgliosis, astrogliosis, and brain atrophy in $Trem2^{-/-}$PS19 mice are greatly attenuated compared with $Trem2^{+/+}$PS19 animals.
Trem2 KO (JAX)	Trem2: Knock-Out	No data..
Trem2 KO (KOMP)	Trem2: Knock-Out	No data.
Trem2 KO (KOMP) x APPPS1	Trem2: Knock-Out; APP: Transgenic; PSEN1: Transgenic	Reduced plaque burden at early stages of plaque deposition but increased plaque burden at later stages, fewer plaque-associated myeloid cells and astrocytes, less phospho-tau in plaque-associated dystrophic neurites, compared with APPPS1.

Trem2 KO (KOMP) x htau	Mapt: Knock-Out; MAPT: Transgenic; Trem2: Knock-Out	Tau phosphorylation and aggregation in the cortex are enhanced in htau mice lacking TREM2, but reactive microglia are smaller and their processes have fewer branches.
Trem2 R47H KI (Haass)	Trem2: Knock-In	Unknown.
Trem2 R47H KI (JAX)	Trem2: Knock-In	Unknown.
Trem2 R47H KI (Lamb/Landreth)	Trem2: Knock-In	No 6E10- or Thioflavin S-positive amyloid plaques were observed at 4 months of age.
Trem2 R47H KI (Lamb/Landreth) X APPPS1-21	Trem2: Knock-In; APP: Transgenic; PSEN1: Transgenic	Reduction in the number and burden of fibrillar amyloid plaques in the hippocampus, fewer plaque-associated myeloid cells, and worse plaque-associated neuritic dystrophy, compared with APPPS1-21 mice homozygous for wild-type Trem2.
Trem2 R47H KI x APOE4	APOE: Knock-In; Trem2: Knock-In	No data.
Ts65Dn	Other	Brain is grossly normal. Age-dependent cholinergic neurodegeneration and reduced NGF in the basal forebrain. Age-related elevation of APP and Aβ in the hippocampus but no β-amyloid pathology.
WSB.APP/PS1	APP: Transgenic; PSEN1: Transgenic	Amyloid plaques, plaque-associated gliosis, cerebral amyloid angiopathy; possible neuron loss in cortex and hippocampal area CA1 in females.
